# A phylogenetic assessment of the polyphyletic nature and intraspecific color polymorphism in the *Bactrocera dorsalis* complex (Diptera, Tephritidae)

**DOI:** 10.3897/zookeys.540.9786

**Published:** 2015-11-26

**Authors:** Luc Leblanc, Michael San Jose, Norman Barr, Daniel Rubinoff

**Affiliations:** 1 Department of Plant and Environmental Protection Sciences, College of Tropical Agriculture and Human Resources, University of Hawaii, 3050 Maile Way, Room 310, Honolulu, Hawaii 96822-2271; 2 United States Department of Agriculture, Animal and Plant Health Inspection Service, Plant Protection and Quarantine, 22675 N. Moorefield Rd., Moore Air Base Building S-6414, Edinburg Texas, 78541; 3 Department of Plant, Soil and Entomological Sciences, University of Idaho, 875 Perimeter Drive MS 2339, Moscow, ID 83844-2339, USA

**Keywords:** *Bactrocera*, *dorsalis*, intraspecific variation, phylogenetics

## Abstract

The *Bactrocera dorsalis* complex (Tephritidae) comprises 85 species of fruit flies, including five highly destructive polyphagous fruit pests. Despite significant work on a few key pest species within the complex, little has been published on the majority of non-economic species in the complex, other than basic descriptions and illustrations of single specimens regarded as typical representatives. To elucidate the species relationships within the *Bactrocera dorsalis* complex, we used 159 sequences from one mitochondrial (*COI*) and two nuclear (*elongation factor-1α* and *period*) genes to construct a phylogeny containing 20 described species from within the complex, four additional species that may be new to science, and 26 other species from *Bactrocera* and its sister genus *Dacus*. The resulting concatenated phylogeny revealed that most of the species placed in the complex appear to be unrelated, emerging across numerous clades. This suggests that they were placed in the *Bactrocera dorsalis* complex based on the similarity of convergent characters, which does not appear to be diagnostic. Variations in scutum and abdomen color patterns within each of the non-economic species are presented and demonstrate that distantly-related, cryptic species overlap greatly in traditional morphological color patterns used to separate them in keys. Some of these species may not be distinguishable with confidence by means other than DNA data.

## Introduction

Most of the Dacine fruit flies (Tephritidae: Dacini) are in the genera *Bactrocera* (651 described species) and *Dacus* (270 species), with many species (73 *Bactrocera* and 11 *Dacus*) bred from commercial/edible fruit and fleshy vegetables (Vargas et al. 2015). Species of *Bactrocera* thrive in the endemic rainforest habitats of South-East Asia and Australasia, with a high degree of host specialization and a large number of cryptic species (Drew and Hancock 2000, Drew 2004).

Among the pest species, *Bactrocera dorsalis* (Hendel) (= the Oriental fruit fly) is the most destructive and polyphagous species (Vargas et al. 2015), belonging to a large complex of similar-looking species: the *Bactrocera dorsalis* complex (hereafter referred to as the OFF complex). The first reference to the OFF complex was by Hardy (1969), who recognized and provided a key to *Bactrocera dorsalis* and 15 other non-economic species. Subsequently, Drew and Hancock (1994) revised the group from South-East Asia, describing 40 new species and splitting *Bactrocera dorsalis* into four distinct species, resulting in a total of 52 species, plus 16 species in Australasia (Drew 1989). Among the combination of character states defining the complex, they included a mostly black scutum and abdomen terga III–V with a medial longitudinal band forming a “T-shaped” pattern with the transverse band at base of tergum III, and with variable dark patterns on lateral margins of terga III–V. Currently, 85 species are recognized, taking into account the recent revision (Drew and Romig 2013) and synonymization (Schutze et al. 2014). Six of the species (*Bactrocera carambolae* Drew & Hancock, *Bactrocera caryeae* (Kapoor), *Bactrocera dorsalis*, *Bactrocera kandiensis* Drew & Hancock, *Bactrocera occipitalis* (Bezzi), and *Bactrocera trivialis* (Drew)) in the complex are significant pests of cultivated fruit (Vargas et al. 2015).

While literature abounds on the taxonomy, genetic diversity, biology and management of the economic species (Clarke et al. 2005, Schutze et al. 2014a), very little is known about most of the other species in the OFF complex, other than basic taxonomic descriptions. Identification to species level is challenging for many species, due to uniform appearance and extensive intraspecific morphological variation. Morphological diagnostic tools were developed for the economic species, based of wing morphometrics (Schutze et al. 2012) and ovipositor and aedeagus lengths (Iwaizumi et al. 1997, Drew et al. 2008, Krosch et al. 2013, White 2000). Some of the species, especially *Bactrocera dorsalis*, display a broad range of color patterns and length of aedeagus and ovipositor, that have resulted in the description of geographic variants as new species, which were subsequently argued to be conspecific (Schutze et al. 2012, 2014b), and synonymized (Schutze et al. 2014a). The range of color variation in the scutum and abdomen was characterized to some extent for *Bactrocera dorsalis* and *Bactrocera carambolae* (e.g. Nishida and Vargas 1992, Iwahashi 1999, Drew et al. 2005, Leblanc et al. 2013), but no information has been published for the other 83 species.

Species descriptions and illustrations in published monographs (Drew 1989, Drew and Hancock 1994, Drew and Romig 2013) are based on the most commonly encountered morphological variants, and little information is presented on intraspecific variation. The dichotomous key in Drew and Hancock (1994) is based on these most common variants, hence difficult to use to identify more atypical specimens. An attempt to account for variation in an interactive CD-ROM key (Lawson et al. 2003) yielded limited success (Clarke et al. 2005). In addition to the described species, there may likely exist cryptic species, hard to distinguish by morphological means, which can be separated with the help of genetic sequencing (e.g. Carew et al. 2011, Dujardin and Kitthawee 2013).

Clarke et al. (2005), when reviewing the data available at the time, stated that phylogenetic studies using limited taxa and genes may not demonstrate the monophyly of the complex. However, recent molecular phylogenies which include the OFF complex have found that most species form a well-defined monophyletic clade (Krosch et al. 2012, Virgilio et al. 2015). However, these studies only included methyl eugenol-attracted species and were limited to six economic species and six, mainly Australian, non-pest species such as *Bactrocera cacuminata* (Hering) and *Bactrocera opiliae* (Drew and Hardy). An alternate, polyphyletic complex was indicated by a phylogeny based on one mitochondrial and two nuclear genes by San Jose et al. (2013), but sampling was limited.

Our goal was to examine the *Bactrocera dorsalis* species complex more broadly than the few frequently targeted pest species. This is accomplished by reporting and analyzing novel molecular and morphological data on 22 non‐pest species in the complex, in the context of the main pest species and selected outgroups. These data are used to: (i) determine through phylogenetic analysis if the complex is monophyletic or polyphyletic; (ii) provide diagnostic molecular data for over 25 species for which such data is currently lacking; and (iii) determine the utility of thoracic and abdominal color/pattern variation as species level diagnostic characters.

## Materials and methods

### Taxa sampling

The molecular phylogenies presented here are based on DNA sequences of 53 specimens collected in Asia, Australia, Oceania, the United States and Africa. These specimens include 47 species of *Bactrocera* belonging to five subgenera (including 24 species from the OFF complex), three species of *Dacus*, and *Ceratitis capitata* (Wiedemann) as the outgroup, (Table 1). In addition, we examined the morphology of thousands of specimens of the economic species and over 1,600 specimens of 22 non-economic species in the OFF complex. Two hundred and thirty seven representatives of these, selected to cover a broad range of color variants, were sequenced for the *COI* gene, as detailed below, to confirm morphological identifications and document intraspecific variation in morphological characters. In addition to examining the color pattern of individual specimens, photographs of the scutum and abdomen were taken, for all the sequenced specimens, and used to compile the variation plates (Figures 2–15). The number of specimens examined and sequenced for individual species are included in the figure captions.

**Table 1.** Species, lure response, collecting locality and voucher code and GenBank accession number for sequences for the species used in this study.

Species	Lure	Locality	Voucher	GenBank Accessions		
***Bactrocera (Bactrocera)***				*COI*	*EF-1α*	*Period*
**Species in *Bactrocera dorsalis* complex**						
*Bactrocera bivittata* Li & Wang	Methyl eugenol	Laos: Luang Namtha	ms1305	KT594878	KT594827	KT594785
*Bactrocera cacuminata* (Hering)	Methyl eugenol	Australia: NSW, Valery	ms1997	KT594887	KT594822	KT594787
*Bactrocera carambolae* Drew & Hancock	Methyl eugenol	Malaysia: Penang, Teluk Bahang	ms1439	KF184076	KF184222	KF184149
*Bactrocera dongnaiae* Drew & Romig	Cue-lure	Cambodia: Koh Kong	ms1109	KT594897	KT594830	KT594789
*Bactrocera dorsalis* (Hendel) (sensu stricto)	Methyl eugenol	Hawaii: Oahu, Makiki	ms0853	KF184084	KF184230	KF184157
*Bactrocera dorsalis* (Hendel) (*Bactrocera invadens*)	Methyl eugenol	Sénégal: Ziguinchor	ms0898	KF184092	KF184238	KF184165
*Bactrocera dorsalis* (Hendel) (*Bactrocera papayae*)	Methyl eugenol	Malaysia: Penang, Teluk Bahang	ms1428	KF184067	KF184213	KF184140
*Bactrocera fuscitibia* Drew & Hancock	Cue-lure	Cambodia: Koh Kong	ms1175	KT594899	KT594831	KT594790
*Bactrocera kanchanaburi* Drew & Hancock	Methyl eugenol	Cambodia: Koh Kong	ms1300	KT594905	KT594833	KT594792
*Bactrocera kohkongiae* Leblanc	Cue-lure	Cambodia: Koh Kong	ms1139	KT591145	KT591136	KT591129
*Bactrocera laithieuiae* Drew & Romig	Cue-lure	Cambodia: Koh Kong	ms3762	KT594916	KT594823	KT594793
*Bactrocera latilineola* Drew & Hancock	Methyl eugenol	Cambodia: Koh Kong	ms1114	KT594917	KT594834	KT594794
*Bactrocera lombokensis* Drew & Hancock	Cue-lure	Laos: Luang Namtha	ms1548	KT594922	KT594836	
*Bactrocera melastomatos* Drew & Hancock	Cue-lure	Malaysia: Kedah, Mount Jerai	ms1411	KT594924	KT594837	KT594796
*Bactrocera occipitalis* (Bezzi)	Methyl eugenol	Philippines: Los Baños	ms1985	KT594931	KT594824	KT594798
*Bactrocera osbeckiae* Drew & Hancock	Cue-lure	Cambodia: Koh Kong	ms1163	KT594938	KT594841	KT594801
*Bactrocera paraarecae* Drew & Romig	Methyl eugenol	Laos: Luang Namtha	ms1110	KF184040	KF184186	KF184113
*Bactrocera propinqua* (Hardy & Adachi)	Cue-lure	Laos: Luang Namtha	ms1167	KF184053	KF184199	KF184126
*Bactrocera quasiinfulata* Drew & Romig	Cue-lure	Laos: Luang Namtha	ms1546	KT594970	KT594843	KT594803
*Bactrocera raiensis* Drew & Hancock	Methyl eugenol	Laos: Luang Namtha	ms1331	KT594972	KT594844	KT594804
*Bactrocera thailandica* Drew & Hancock	Cue-lure	Thailand: Chiang Mai	ms1047	KT594985	KT594852	KT594812
*Bactrocera usitata* Drew & Hancock	Cue-lure	Cambodia: Koh Kong	ms1173	KT594999	KT594854	KT594814
*Bactrocera* species 54	Cue-lure	Thailand: Chiang Mai	ms1182	KT594976	KT594847	KT594807
*Bactrocera* species 55	Cue-lure	Laos: Luang Namtha	ms1181	KT594979	KT594848	KT594808
*Bactrocera* species 59	Cue-lure	Laos: Luang Namtha	ms1164	KT594981	KT594849	KT594809
*Bactrocera* species 60	Methyl eugenol	China: Jinghong	ms3633	KT594982	KT594850	KT594810
**Other species**						
*Bactrocera aethriobasis* (Hardy)	Methyl eugenol	Cambodia: Koh Kong	ms1557	KT594862	KT594825	KT594783
*Bactrocera albistrigata* deMeijere	Cue-lure	Malaysia: Penang, Teluk Bahang	ms1395	KT594863	KT594826	KT594784
*Bactrocera bhutaniae* Drew & Romig	Cue-lure	Laos: Luang Namtha	ms1166	KF184052	KF184198	KF184125
*Bactrocera bryoniae* (Tryon)	Cue-lure	Australia: Bundaberg	ms1515	KT594886	KT594828	KT594786
*Bactrocera correcta* (Bezzi)	Methyl eugenol	Cambodia: Koh Kong	ms1093	KT594896	KT594829	KT594788
*Bactrocera kirki* (Froggatt)	Cue-lure	French Polynesia: Tahiti	ms0894	KF184090	KF184236	KF184163
*Bactrocera latifrons* (Hendel)	Latilure/cade oil	Hawaii: Oahu	ms0882	KF184085	KF184231	KF184158
*Bactrocera limbifera* (Bezzi)	Cue-lure	Cambodia: Koh Kong	ms1108	KT594921	KT594835	KT594795
*Bactrocera nigrotibialis* (Perkins)	Cue-lure	Cambodia: Koh Kong	ms1033	KT594930	KT594838	KT594797
*Bactrocera ochrosiae* (Malloch)	Cue-lure	Mariana Islands: Saipan	ms1485	KT594932	KT594839	KT594799
*Bactrocera paradiospyri* Chen, Zhou & Li	Methyl eugenol	Thailand: Chiang Mai	ms1470	KT594956	KT594842	KT594802
*Bactrocera rubigina* (Wang & Zhao)	Cue-lure	China: Jinghong	ms3544	KT594974	KT594845	KT594805
*Bactrocera tryoni* (Froggatt)	Cue-lure	French Polynesia: Tahiti	ms0892	KF184088	KF184234	KF184161
*Bactrocera tuberculata* (Bezzi)	Methyl eugenol	Thailand: Chiang Mai	ms1083	KT594998	KT594853	KT594813
*Bactrocera umbrosa* (Fabricius)	Methyl eugenol	Cambodia: Koh Kong	ms1002	KF184032	KF184178	KF184105
*Bactrocera wuzhishana* Li & Wang	Methyl eugenol	Thailand: Chiang Mai	ms1070	KT595000	KT594855	KT594815
*Bactrocera zonata* (Saunders)	Methyl eugenol	Thailand: Chiang Mai	ms1559	KT595002	KT594857	KT594817
***Bactrocera (Daculus)***						
*Bactrocera oleae* (Gmelin)	No lure	USA: California	ms1387	KT594933	KT594840	KT594800
***Bactrocera (Notodacus)***						
*Bactrocera xanthodes* (Broun)	Methyl Eugenol	French Polynesia: Rurutu	ms0896	KT595001	KT594856	KT594816
***Bactrocera (Sinodacus)***						
*Bactrocera hochii* (Zia)	Cue-lure	Laos: Luang Namtha	ms1369	KT594904	KT594832	KT594791
***Bactrocera (Zeugodacus)***						
*Bactrocera cucurbitae* (Coquillett)	Cue-lure	Cambodia: Koh Kong	ms0987	KF184104	KF184250	KF184177
*Bactrocera scutellaris* (Bezzi)	Cue-lure	Thailand: Chiang Mai	ms1030	KT594975	KT594846	KT594806
*Bactrocera tau* (Walker)	Cue-lure	Laos: Luang Namtha	ms1006	KT594984	KT594851	KT594811
**Genus *Dacus***						
Dacus (Didacus) ciliatus Loew	None	South Africa: Stellenbosch	ms1576	KT595004	KT594859	KT594819
Dacus (Psilodacus) pullescens Munro	None	South Africa: Calitzdorp	ms1578	KT595005	KT594860	KT594820
Dacus (Mellesis) sinensis Wang	Cue-lure	Laos: Luang Namtha	ms1372	KT595006	KT594861	KT594821
**Genus *Ceratitis***						
*Ceratitis capitata* (Wiedemann)	Trimedlure	Hawaii: Oahu	ms0865	KT595003	KT594858	KT594818

### Validation of identification

Our specimens in the OFF complex were initially tentatively identified to species using available resources (Drew and Hancock 1994, Lawson et al. 2003, Drew and Romig 2013). These determinations were then confirmed by comparing pinned representatives and photographic plates of color variation to the large series of specimens used to produce the above publications, deposited in the Queensland Department of Agriculture and Fisheries (QDAF) insect collection (Ecosciences Precinct, Brisbane). The identifications were also confirmed by R.A.I. Drew, an expert on *Bactrocera* morphology. Species referred to by numbers in previous publications (San Jose et al. 2013, Leblanc et al. 2013, 2014) and included in this study were identified as *Bactrocera osbeckiae* Drew and Hancock (species 22), *Bactrocera bhutaniae* Drew and Romig (species 25), *Bactrocera paraarecae* (species 26), and *Bactrocera propinqua* (Hardy and Adachi) (species 45).

### DNA extraction, amplification, and sequencing

For each specimen, one to three legs were used for total genomic DNA extraction. The remainder of the specimen was deposited as a voucher in the University of Hawaii Insect Museum (UHIM) for preservation and morphological studies (Table 1). Genomic DNA was extracted using the DNeasy animal blood and tissue extraction kit following manufacturer’s protocol (Qiagen, Inc., Valencia, CA). Three different gene regions were amplified: the mitochondrial gene *cytochrome c oxidase I* (*COI*, 780 bp) and the nuclear genes, *elongation factor-1α* (*EF-1α*, 759 bp) and *period* (*PER*, 450 bp). These three genes were selected because each has been demonstrated to be informative in distinguishing populations, species complexes, species, or genera in Diptera (Folmer et al. 1994, Simon et al. 1994, Cho et al. 1995, Bauzer et al. 2002, Moulton and Wiegmann 2004, Barr et al. 2005, Foley et al. 2007, Virgilio et al. 2009, Gibson et al. 2011, San Jose et al. 2013). Gene amplification followed San Jose et al. (2013). All polymerase chain reaction (PCR) products were visualized on 1% agarose gel and purified using QIAquick spin columns (Qiagen, Inc.) according to the manufacturer’s protocol. Bidirectional DNA sequencing was performed at the Advanced Studies of Genomics, Proteomics and Bioinformatics (ASGPB) sequencing facility of the University of Hawaii at Manoa (http://asgpb.mhpcc.hawaii.edu/).

### Sequence alignment, nucleotide composition, and phylogenetic analysis

Sequence alignments were performed with the software package Geneious 7.1.7 (Biomatters ltd.). Heterozygosity in the nuclear genes was present in most samples. Ambiguity codes (i.e., notation according to International Union of Pure and Applied Chemistry (IUPAC)) were used to denote heterozygous base pairs, and these codes were used in the subsequent analysis. Sequence alignment for each gene was conducted in Geneious using the Muscle option with default settings (Edgar 2004). We used jModeltest and the Akaike information criterion (Darriba et al. 2012) to determine the most appropriate evolutionary model for each gene in our analysis. Phylogenetic analyses were performed with both Maximum Likelihood and Bayesian Inference. MrBayes 3.2.1 (Ronquist et al. 2012) was used for Bayesian analyses and RaxML (Stamatakis et al. 2008) was used for maximum likelihood (ML). We used jModeltest (Darriba et al. 2012) to determine the most appropriate model for each partition. We concatenated our datasets by gene and used a GTR+ Γ model for each gene in the Bayesian analysis general time reversible model (Tavaré 1986) with gamma distribution of rates (GTRGAMMA) for each gene in our likelihood analysis. We first analyzed each gene separately and subsequently concatenated them into a single dataset partitioned, by gene, using Maximum Likelihood and Bayesian inference. For each individual gene analysis (*COI*, *period*, and *EF-1α*) we ran four independent Bayesian runs in MrBayes 3.2.1 using the default settings. Each run started from a random tree using default priors sampling every one thousand generation for 10 million generations with a relative burn-in of 25%. We used the program Tracer 1.5 (Rambaut and Drummond 2009) to assess convergence of standard deviation in variance for Bayesian analyses. For RaxML analyses, each dataset included 10 ML tree searches with default settings, using a random starting tree to find the tree with the best likelihood score. One thousand Maximum Likelihood bootstrap replicates were conducted in Raxml to assess support for inferred relationships. For the concatenated dataset, we partitioned the data by gene and ran MrBayes using the same settings as the individual gene analyses except the parameters statefreq, revmat, shape, and pinvar were unlinked between partitions. For the Maximum Likelihood analysis of the partitioned concatenated dataset, we ran RaxML using the same settings and analyses for each partition as when genes were analyzed individually. Trees were visualized using FigTree v1.4.0 (Rambaut 2012) and rooted with *Ceratitis capitata*. *COI* sequences for all non-economic species in the *Bactrocera dorsalis* complex for which at least four sequences were available were analyzed using the program DNAsp to provide basic population genetic variability summary statistics (*Hn*, *h π*, *S*).

## Data Resources

Sequences listed on Table 1, as well as COI sequences for all specimens included on all figure plates, were deposited into GenBank KT591129 to KT591164 and KT594783 to KT595006.

## Results

**Figure 1.**
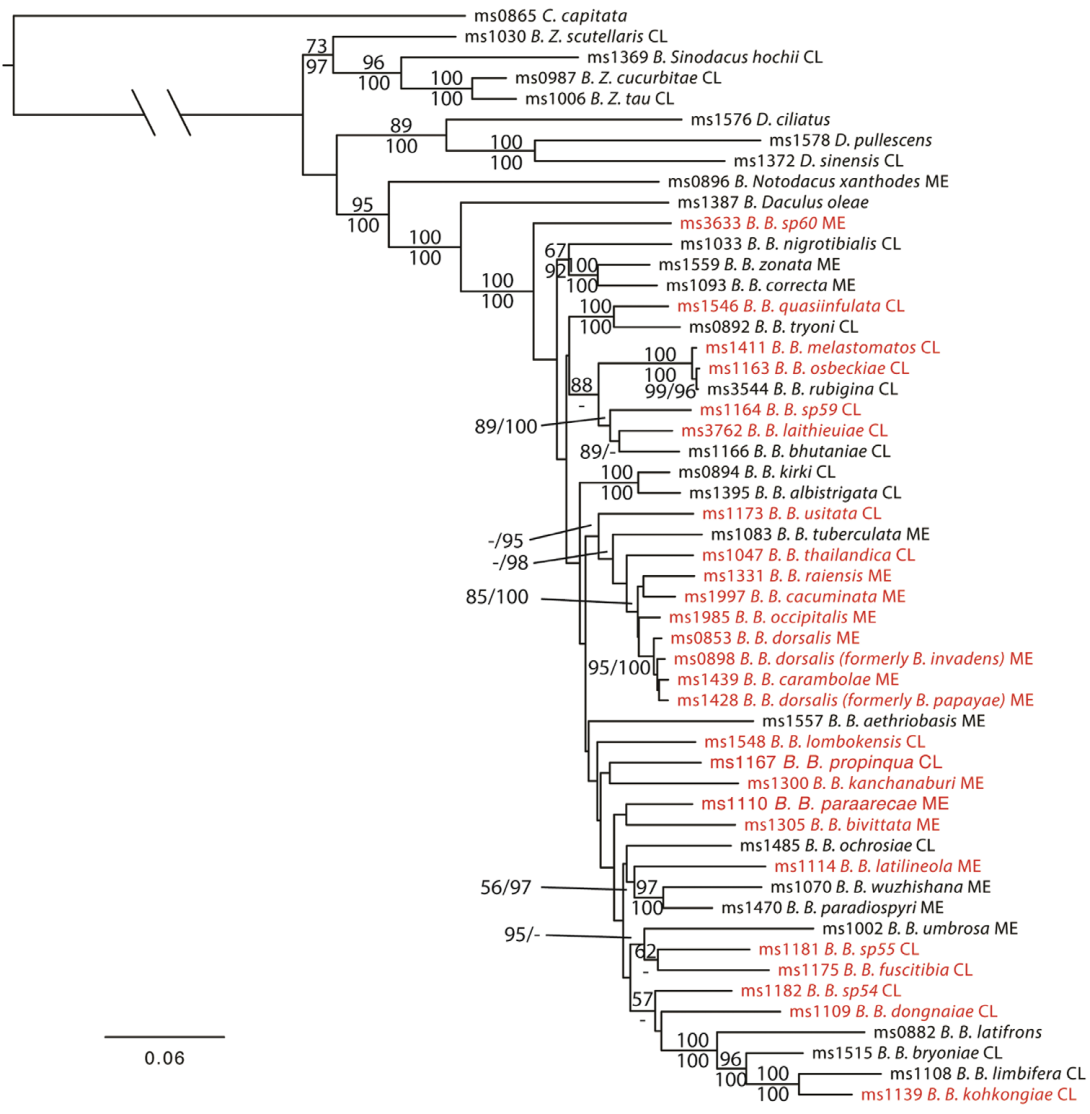
Maximum likelihood tree, concatenated, based three gene (*COI, period, EF-1α*) dataset. Support values above branches are Maximum Likelihood Bootstrap values / Bayesian Posterior Probabilities. Scale bar indicates the number of substitutions per site. Species in the Oriental fruit fly complex are outlined in red.

Topological differences between the individual gene trees were not supported with high bootstrap values and posterior probabilities (<50% BS <0.9 PP) and overall individual gene trees were poorly resolved, with *COI* providing more signal for the more recent divergences (Suppl. material 1) and the nuclear genes providing signal for deeper relationships (Suppl. material 2, 3). However the concatenated analysis produced a well-resolved tree (Figure 1) which is consistent with previous studies (Krosch et al. 2012, San Jose et al. 2013, Virgilio et al. 2015). In the concatenated phylogeny, the *Zeugodacus* group of subgenera (as defined by Drew and Hancock 2000) is sister to *Dacus* and the *Bactrocera*+*Notodacus*+*Daculus* clades, which themselves are sister taxa. This renders *Bactrocera* paraphyletic with respect to *Dacus*, as suggested previously (White 2006, Krosch et al. 2012, Virgilio et al. 2015). However the relationship is not strongly supported in the tree and additional genes and taxa are necessary to fully resolve this relationship. The subgenus *Bactrocera* is monophyletic in the concatenated phylogeny (100 BS, 1.0 PP). The inclusion of many non-economic OFF complex species in our study shows with high support that despite a similar appearance, the complex is a highly polyphyletic group. Multiple, well-supported, clades (75–100% BS values) in the subgenus *Bactrocera* contain a mix of species previously thought to belong to the OFF complex and non-OFF complex species. One clear example is the inclusion of non-OFF complex *Bactrocera bryoniae*, *Bactrocera latifrons*, *Bactrocera limbifera*, with *Bactrocera kohkongiae*, which fits in the OFF complex (Figure 15 A–C) in a strongly supported (100%, 100% PP value) clade (Figure 1). This indicates that, despite low support for the backbone topology in the subgenus *Bactrocera*, the polyphyletic nature of the OFF complex is still well supported. The main pest species in the complex (*Bactrocera carambolae* and *Bactrocera dorsalis*, now including *Bactrocera papayae* and *Bactrocera invadens*, see Schutze et al. 2014a) form a monophyletic unit with very little genetic differentiation (<1.3% in *COI*) between them, and rest within a well defined clade that includes several other species attracted to methyl eugenol (*Bactrocera occipitalis*, *Bactrocera cacuminata*, *Bactrocera raiensis*). Three species, *Bactrocera melastomatos* (Figure 9F–O), *Bactrocera osbeckiae* (Figure 10) and *Bactrocera rubigina* (Figure 14F), were genetically indistinguishable using *COI* (0.1% pair-wise difference) in the phylogeny, appearing together in a single lineage, despite having very distinctive color patterns. Interestingly, they were slightly more distinct in the nuclear genes (1.1% *EF-1α* and 1% *period* pair-wise difference), which was not the case for most species. Population genetic statistics, based on *COI* sequences, showed high levels of haplotype diversity for most of the non-economic species in the *Bactrocera dorsalis* complex (Table 2).

**Table 2.** Summary statistics of genetic variability, based on *COI* gene sequences, for non-economic species in the *Bactrocera dorsalis* complex.

Species	Sample size	Haplotypes (Nh)	Haplotype diversity (h)	Nucleotide diversity (pi)	Segregating sites (s)
*Bactrocera bhutaniae*	33	25	0.966	0.02557	86
*Bactrocera bivittata*	10	9	0.978	0.00320	10
*Bactrocera cacuminata*	11	4	0.491	0.00161	5
*Bactrocera fuscitibia*	6	5	0.933	0.00676	14
*Bactrocera kanchanaburi*	15	13	0.981	0.00768	31
*Bactrocera kohkongiae*	22	17	0.952	0.00472	23
*Bactrocera latilineola*	4	3	0.833	0.00320	5
*Bactrocera melastomatos*	8	4	0.643	0.00127	4
*Bactrocera osbeckiae*	35	13	0.704	0.00717	18
*Bactrocera paraarecae*	5	5	1.000	0.01536	29
*Bactrocera propinqua*	24	23	0.996	0.01047	40
*Bactrocera thailandica*	56	13	0.386	0.00145	24
*Bactrocera usitata*	5	5	1.000	0.01076	17

**Figure 2.**
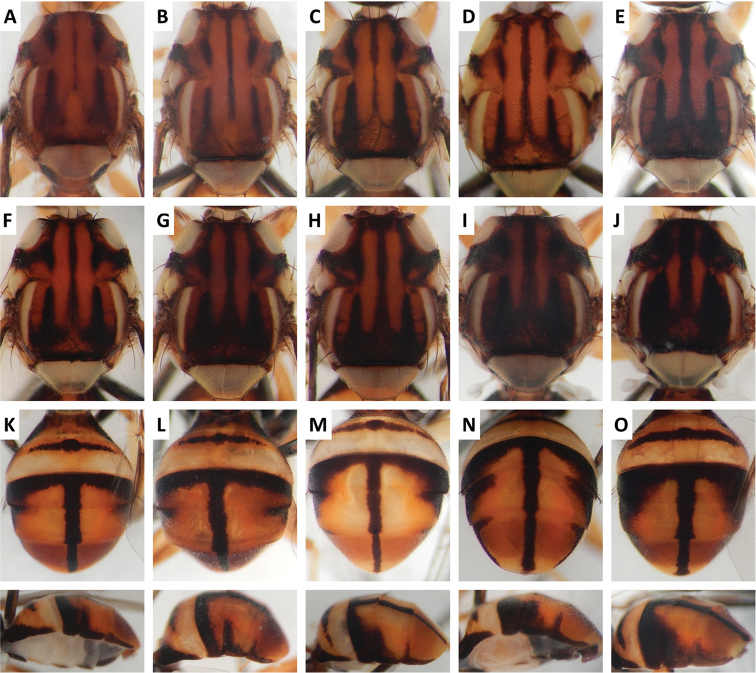
Variation in color pattern of scutum and abdomen in *Bactrocera bhutaniae* Drew and Romig (321 specimens examined and 36 sequenced). Voucher codes are: **A** ms3593 **B** ms3531 **C** ms3533 **D** ms4321 **E** ms2034 **F** ms1166 **G** ms2031 **H** ms3527 **I** ms3580 **J** ms1168 **K** ms2030 **L** ms3578 **M** ms4329 **N** ms3527 **O** ms1168.

**Figure 3.**
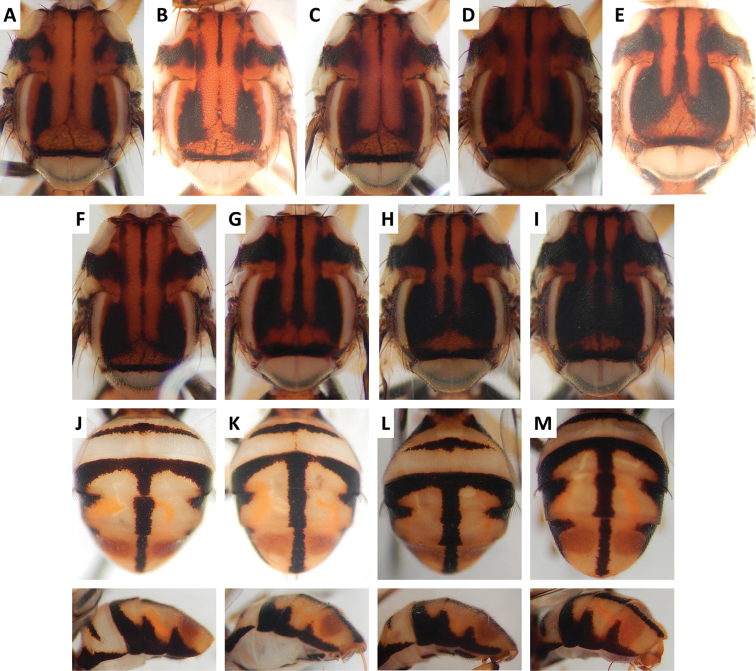
Variation in color pattern of scutum and abdomen in *Bactrocera bivittata* Li and Wang (47 specimens examined and 10 sequenced). Voucher codes are: **A** ms3606 **B** ms1305 **C** ms1304 **D** ms3607 **E** ms3605 **F** ms3604 **G** ms3609 **H** ms3608 **I** ms1790 **J** ms3605 **K** ms3606 **L** ms3609 **M** ms3604.

**Figure 4.**
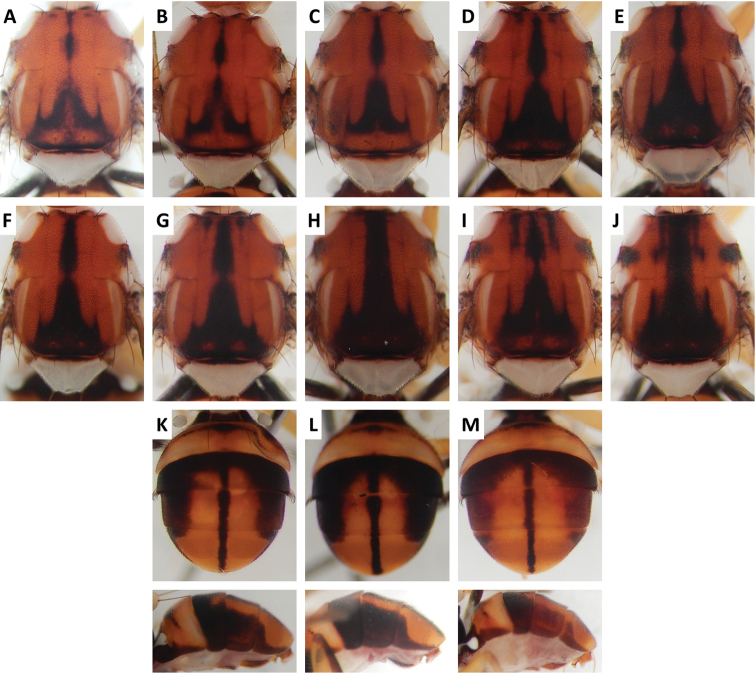
Variation in color pattern of scutum and abdomen in *Bactrocera cacuminata* (Hering) (> 300 specimens examined and 12 sequenced). Voucher codes are: **A** ms2003 **B** ms2005 **C** ms1998 **D** ms2008 **E** ms1999 **F** ms1997 **G** ms2010 **H** ms2004 **I** ms2009 **J** ms2002 **K** ms2005 **L** ms2008 **M** ms2009.

**Figure 5.**
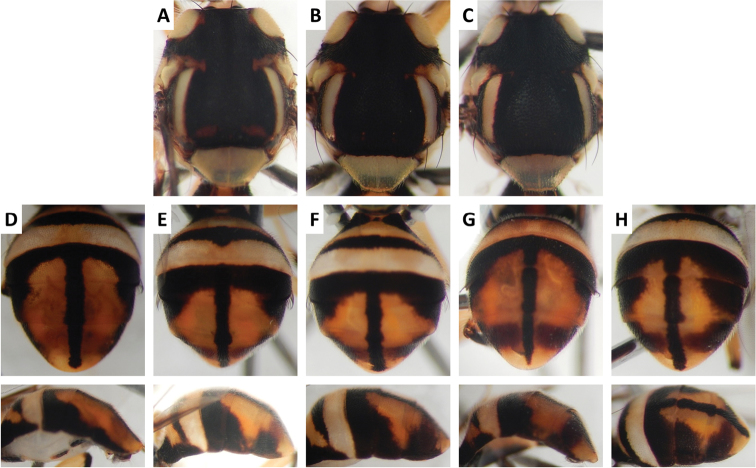
Variation in color pattern of scutum and abdomen in *Bactrocera fuscitibia* (Drew and Hancock) (33 specimens examined and 6 sequenced). Voucher codes are: **A** ms1178 **B** ms1177 **C** ms1297 **D** ms1175 **E** ms1176 **F** ms1177 **G** ms1178 **H** ms1297.

**Figure 6.**
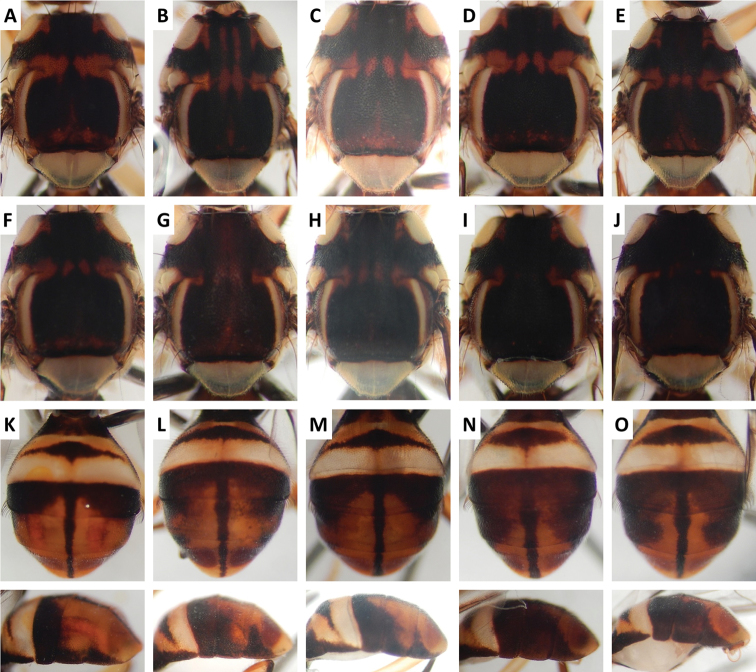
Variation in color pattern of scutum and abdomen in *Bactrocera kanchanaburi* Drew and Hancock (47 specimens examined and 16 sequenced). Voucher codes are: **A** ms3599 **B** ms1300 **C** ms3598 **D** ms1303 **E** ms3725 **F** ms1302 **G** ms3597 **H** ms3596 **I** ms3728 **J** ms3603 **K** ms3599 **L** ms3728 **M** ms1300 **N** ms1301 **O** ms3729.

**Figure 7.**
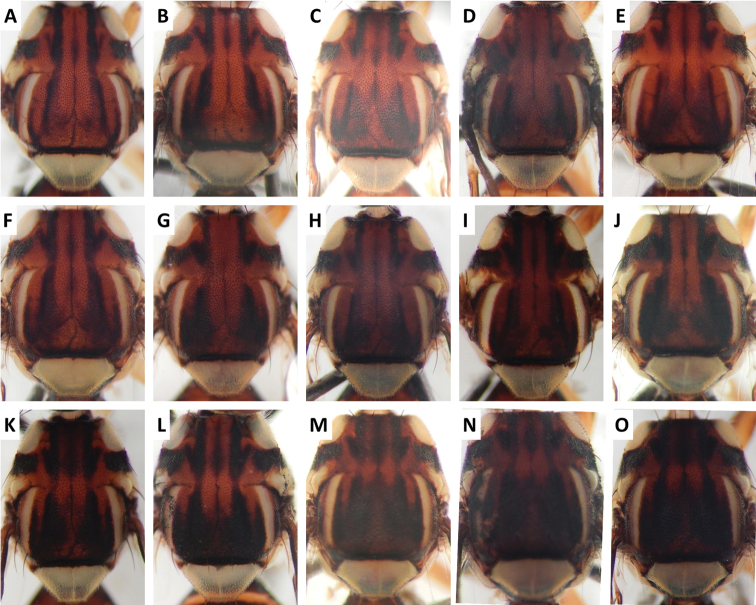
Variation in color pattern of scutum in *Bactrocera kohkongiae* Leblanc (210 specimens examined and 22 sequenced). Voucher codes are: **A** ms1149 **B** ms1144 **C** ms1142 **D** ms1780 **E** ms1148 **F** ms1145 **G** ms1307 **H** ms1143 **I** ms1141 **J** ms1146 **K** ms1151 **L** ms1785 **M** ms1140 **N** ms1781 **O** ms1150.

**Figure 8.**
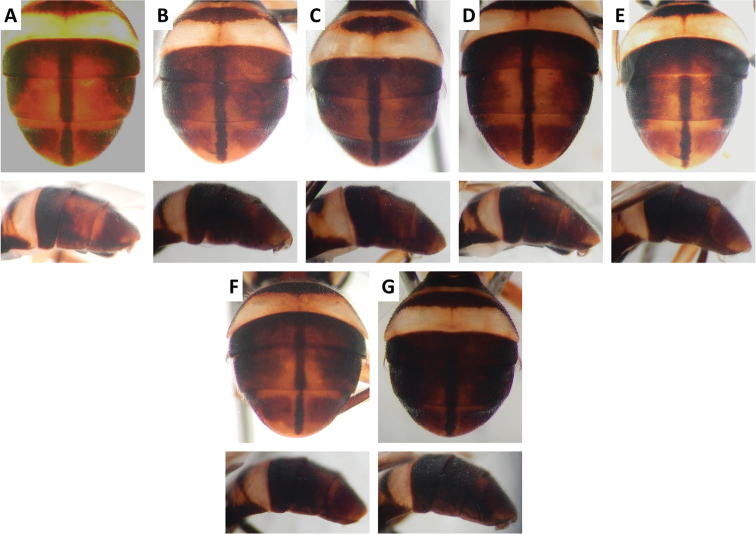
Variation in color pattern of abdomen in *Bactrocera kohkongiae* Leblanc. Voucher codes are: **A** ms1149 **B** ms1147 **C** ms1145 **D** ms1785 **E** ms1146 **F** ms1139 **G** ms1137.

**Figure 9.**
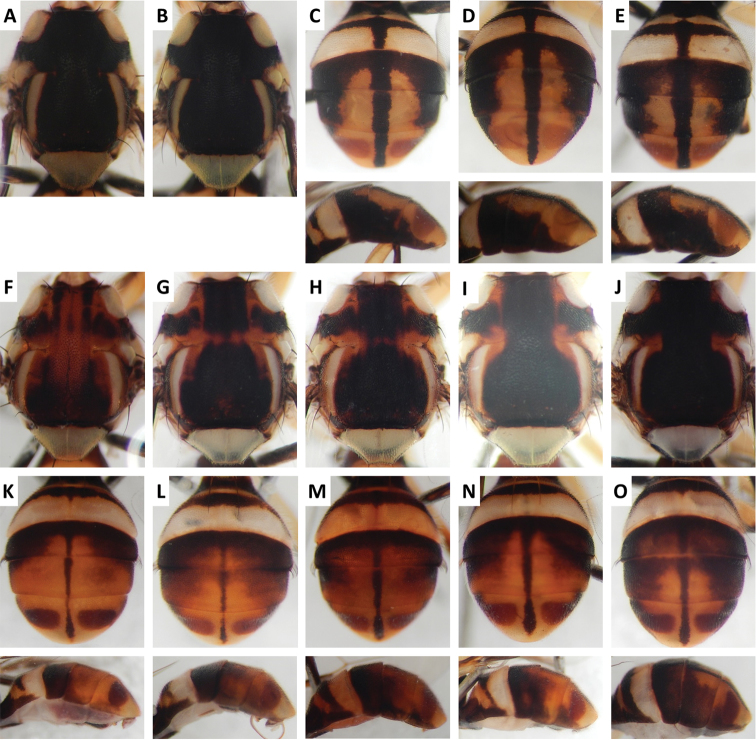
Variation in color pattern of scutum and abdomen in *Bactrocera latilineola* Drew and Hancock (**A–E**) (11 specimens examined and 4 sequenced) and *Bactrocera melastomatos* Drew and Hancock (**F–O**) (46 specimens examined and 8 sequenced). Voucher codes are: **A** ms1114 **B** ms2025 **C** ms2025 **D** ms2024 **E** ms1299 **F** ms1415 **G** ms1416 **H** ms1410 **I** ms1412 **J** ms1411 **K** ms1416 **L** ms1417 **M** ms1413 **N** ms1410 **O** ms1411.

**Figure 10.**
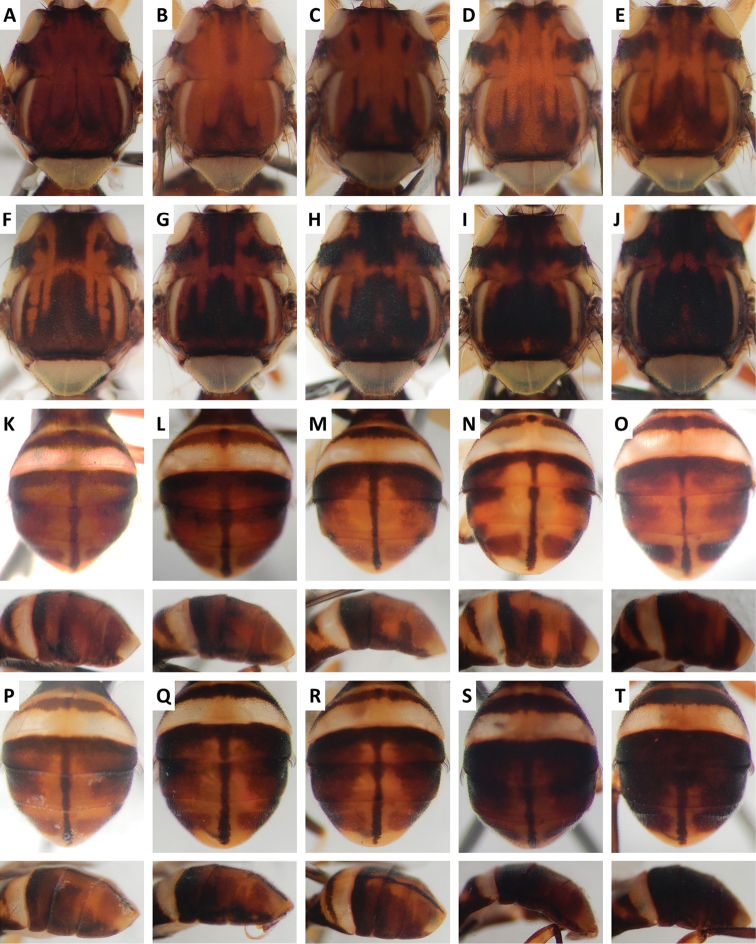
Variation in color pattern of scutum and abdomen in *Bactrocera osbeckiae* Drew and Romig (100 specimens examined and 39 sequenced). Voucher codes are: **A** ms1161 **B** ms3559 **C** ms3558 **D** ms3553 **E** ms3555 **F** ms3561 **G** ms1163 **H** ms3785 **I** ms3764 **J** ms3768 **K** ms1153 **L** ms3758 **M** ms3554 **N** ms1180 **O** ms1138 **P** ms3555 **Q** ms3784 **R** ms3560 **S** ms1154 **T** ms3768.

**Figure 11.**
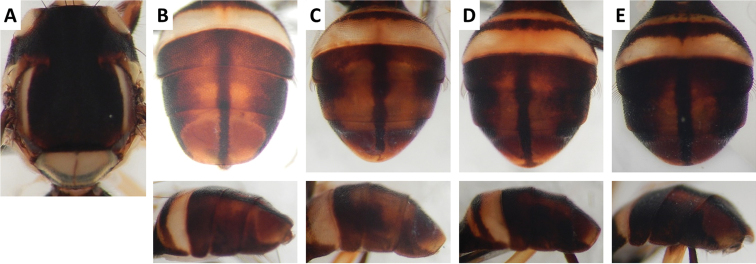
Variation in color pattern of scutum and abdomen in *Bactrocera paraarecae* Drew and Romig (10 specimens examined and 5 sequenced). Voucher codes are: **A** ms1295 **B** ms1296 **C** ms2040 **D** ms1294 **E** ms1110.

**Figure 12.**
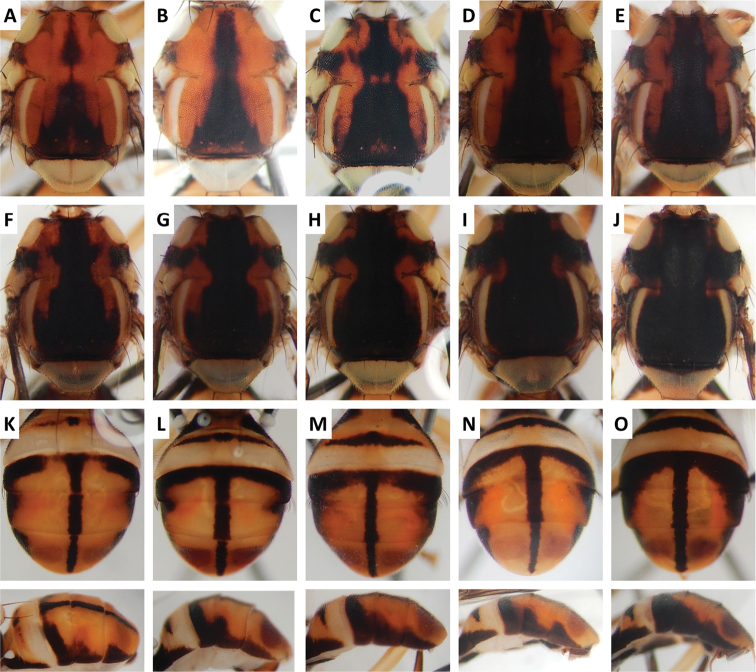
Variation in color pattern of scutum and abdomen in *Bactrocera propinqua* (Hardy and Adachi) (49 specimens examined and 24 sequenced). Voucher codes are: **A** ms4324 **B** ms4331 **C** ms4322 **D** ms3568 **E** ms3571 **F** ms3833 **G** ms3572 **H** ms3567 **I** ms2041 **J** ms1170 **K** ms4331 **L** ms3765 **M** ms3757 **N** ms3572 **O** ms3566.

**Figure 13.**
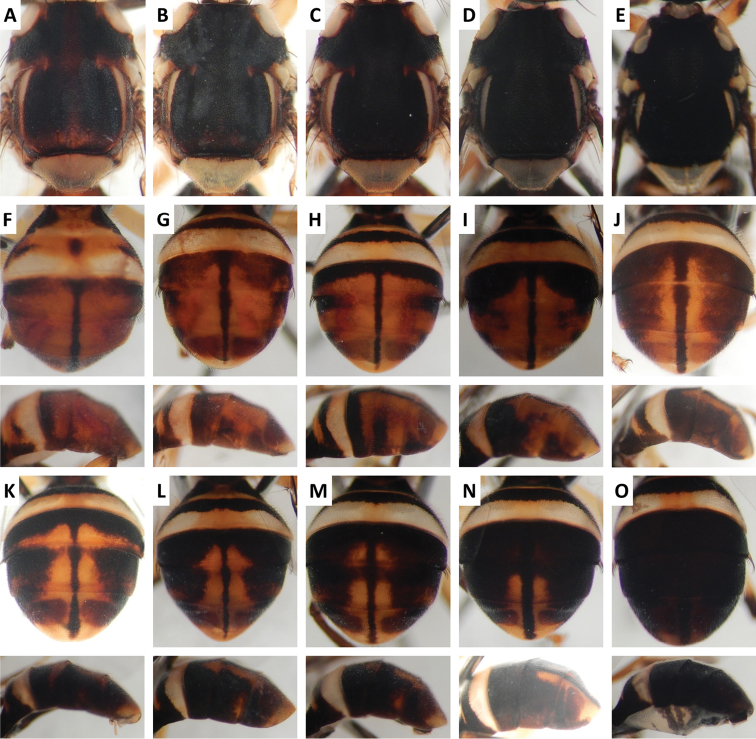
Variation in color pattern of scutum in *Bactrocera thailandica* Drew and Romig (712 specimens and 56 sequenced). Voucher codes are: **A** ms3587 **B** ms3588 **C** ms3586 **D** ms3525 **E** ms1952 **F** ms3576 **G** ms3586 **H** ms3736 **I** ms3585 **J** ms3539 **K** ms3581 **L** ms3538 **M** ms3695 **N** ms3582 **O** ms1949.

**Figure 14.**
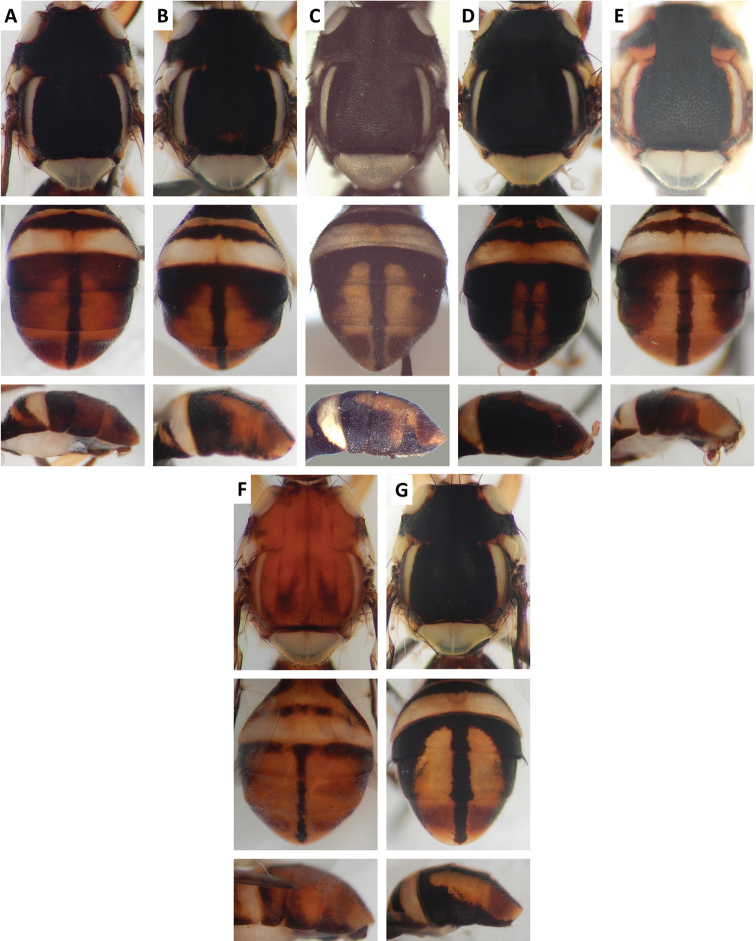
Scutum and abdomen of: **A** *Bactrocera dongnaiae* Drew and Romig (ms1158; 7 specimens examined and 3 sequenced) **B** *Bactrocera laithieuiae* Drew and Romig (ms3762; 1 specimen examined and sequenced) **C** *Bactrocera lombokensis* Drew and Hancock (ms1548; 1 specimen examined and sequenced) **D** *Bactrocera quasiinfulata* Drew and Romig (ms3455; 4 specimens examined and sequenced) **E** *Bactrocera raiensis* Drew and Hancock (ms1331; 2 specimens examined and 1 sequenced) **F** *Bactrocera rubigina* (Wang and Zhao) (ms3543; 259 specimens examined and 27 sequenced) **G** *Bactrocera usitata* Drew and Hancock (ms2039; 27 specimens examined and 6 sequenced).

**Figure 15.**
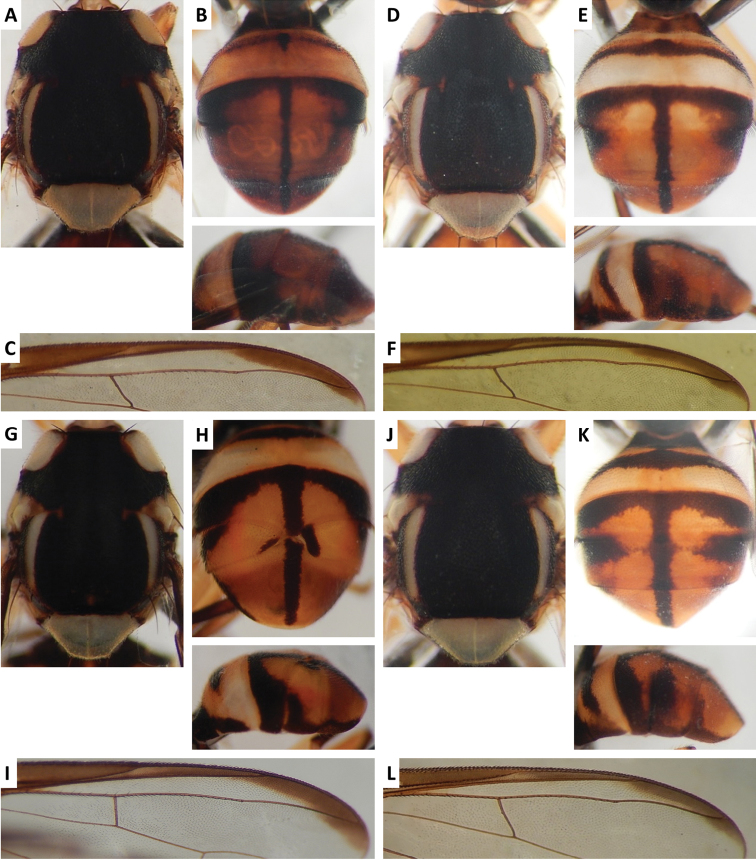
Scutum, abdomen and wing costal region of: **A–C** *Bactrocera* species 54 (ms1798 (wing, scutum), ms3777 (abdomen); 7 specimens examined and sequenced) **D–F** *Bactrocera* species 55 (ms3575; 7 specimens examined and sequenced) **G–I** *Bactrocera* species 59 (ms1164; 1 specimen examined and sequenced) **J–L** *Bactrocera* species 60 (ms3730; 3 specimens examined and sequenced).

Color patterns of scutum and/or abdomen (Figures 2–15) varied extensively within some of the species (Figures 2A–J, 3A–I, 4A–J, 5D–H, 6K–O, 7, 8, 9F–O, 10, 11B–E, 12A–J, 13F–O), and were relatively uniform in others (Figures 2K–O, 3J–M, 4K–M, 5A–C, 6A–J, 9A–E, 11A, 12K–O, 13A–E). Scutum color pattern was highly polymorphic in *Bactrocera bhutaniae* (Figure 2), *Bactrocera bivittata* (Figure 3), *Bactrocera kohkongiae* (Figure 7), *Bactrocera melastomatos* (Figure 9F–O), *Bactrocera osbeckiae* (Figure 10), and *Bactrocera propinqua* (Figure 12). Abdomen pattern was confusingly polymorphic, yet scutum remained uniform in *Bactrocera thailandica* (Figure 13).

Scutum color and variation followed three basic patterns among species for which series of specimens were examined. In *Bactrocera bhutaniae* (Figure 2), *Bactrocera bivittata* (Figure 3), *Bactrocera kohkongiae* (Figure 7), and *Bactrocera osbeckiae* (Figure 10), scutum was predominantly red–brown with a highly variable dark lanceolate pattern. The pattern was composed of a medial and two lateral bands, generally interrupted at the level of the transverse suture, in *Bactrocera bhutaniae* and *Bactrocera bivittata* (medial band usually narrower and lateral bands very broad). The lanceolate pattern was highly variable in *Bactrocera kohkongiae*, from extensively pale with a narrow medial band to almost entirely dark with light markings restricted to the transverse suture, and *Bactrocera osbeckiae*, from mostly dark fuscous, with red-brown markings at level of postpronotal lobes and along transverse suture, to extensive lanceolate red–brown pattern with a broad medial longitudinal band, which can be faint or absent. In *Bactrocera cacuminata* (Figure 4), scutum was red–brown with a single medial dark band widened at apex of scutum and anteriorly narrowed to a point, and with two short lateral bands pointed anteriorly. A similar pattern was frequently observed in *Bactrocera propinqua* (Figure 12), in which the scutum varied from *Bactrocera cacuminata*-like to uniformly dark with light markings at level of transverse suture and inside postpronotal lobes. Scutum was generally uniformly black, with at most small red–brown markings anterior to lateral postsutural vittae, inside postpronotal lobes and sometimes at the level of prescutellar setae, in *Bactrocera fuscitibia* (Figure 5), *Bactrocera latilineola* (Figure 9), *Bactrocera paraarecae* (Figure 11), *Bactrocera thailandica* (Figure 13), and *Bactrocera usitata* (Figure 14H), and frequently with more extensive red–brown markings along transverse suture in *Bactrocera kanchanaburi* (Figure 6) and *Bactrocera melastomatos* (Figure 9 F–J). The shape and width of lateral postsutural vittae was relatively constant for all species except *Bactrocera thailandica* (Figure 13).

Abdomen color for almost all species and variants followed the basic “T-shaped” pattern typical of the *Bactrocera dorsalis* complex, i.e. a black band across the base of tergum III, a narrow to broad medial longitudinal black band covering the entire length of terga III to V, and narrow to broadly expanded lateral black markings on terga III to V. Medial band was broad and lateral markings generally broad along margins of tergum III and narrower on terga IV and V in *Bactrocera bhutaniae* (Figure 2) and *Bactrocera propinqua* (Figure 12), or the markings on terga III and IV expanded and pointed at apex in *Bactrocera bivittata* (Figure 3). Medial band was broad and extended to the base of tergum II and lateral markings broad on terga III, IV, and base of tergum V in *Bactrocera latilineola* (Figure 9C–E). Medial band was narrow (broad in *Bactrocera usitata*) and lateral markings usually broad along terga III–IV and basal half of tergum V in *Bactrocera cacuminata* (Figure 4), *Bactrocera kanchanaburi* (Figure 6), and *Bactrocera usitata* (Figure 14 G). Medial band was broad and lateral markings moderately to very broad on tergum III and IV, and shining spots on tergum V usually black (fuscous to dark fuscous in most other species) and continuous with lateral black markings in *Bactrocera fuscitibia* (Figure 5). Medial band was narrow (broad in *Bactrocera paraarecae*) and lateral markings moderately to very broad but diffuse, rather than well defined (as in previous species), in *Bactrocera kohkongiae* (Figure 8), *Bactrocera melastomatos* (Figure 9 K–O), *Bactrocera osbeckiae* (Figure 10), and *Bactrocera paraarecae* (Figure 11). In *Bactrocera thailandica*, medial band was narrow and the extent of lateral markings varied considerably, from very limited to almost entirely covering the terga except traces of red–brown on tergum V, on either side of medial band (Figure 13).

## Discussion

The concatenated tree demonstrates that the OFF complex is a highly polyphyletic assemblage of unrelated species. Consistent with other published studies, the methyl eugenol responsive *Bactrocera dorsalis*, *Bactrocera carambolae*, *Bactrocera occipitalis*, *Bactrocera cacuminata*, and *Bactrocera raiensis*, form a well-defined monophyletic unit (Krosch et al. 2012, San Jose et al. 2013, Boykin et al. 2013, Virgilio et al. 2015). Because the phylogeny is based on a relatively limited (24%) proportion of all the species included in the OFF complex, adding more species and using multiple genes may reveal scattered clusters of related species, but the proportion of unrelated clades including OFF complex species is likely to remain high.

The widespread conformity of unrelated species to the *dorsalis*-like appearance is unclear. Color patterns in Dacine fruit flies are assumed to mimic wasps (White 2000), though few actual wasp mimic examples exist in Dacine fruit flies, and the OFF complex appearance is not a particularly convincing wasp imitation when compared to other groups of mimics (sesiid moths, syrphid flies, etc.). Whether the similarity represents convergent evolution or a retained ancestral state requires further investigation.

Except for a handful of well-studied species (e.g. *Bactrocera dorsalis*, *Bactrocera carambolae*, *Bactrocera cacuminata*), the definitions and concepts for the majority of the OFF complex species were based on morphology (mainly color patterns), lure response, and generally limited host fruit records. Only now are we starting to better characterize these species with molecular tools. Most of the non-economic species described by Drew and Hancock (1994) included in our study appear to be valid, confirmed by molecular data and comparison of morphological intraspecific variation with large series of specimens in QDAF (L.L., unpublished observations).

Attraction of *Bactrocera osbeckiae* to cue-lure is a new lure record. Morphological variation in our cue-lure trapped specimens (Figure 10) closely matched that observed in the QDAF and Bishop Museum (Honolulu, Hawaii, USA) series, which consist of host-reared specimens without male lure records.

Four species, consistent in appearance with the definition of the OFF complex, could not clearly be identified and are referred to here as numbered species. Species 54 (from Chiang Mai, Thailand) and 55 (Luang Nam Tha, Laos and Jinghong, China) look very similar (Figure 15A–F), yet are genetically distinct (8.85% *COI* pair-wise difference). They both key to *Bactrocera irvingiae* in Drew and Hancock (1994), but neither can be confidently matched to that species, even after comparison with series of pinned specimens of *Bactrocera irvingiae* and other OFF complex species in the QDAF collection. Also, *Bactrocera irvingiae* was collected further south in Thailand (Khao Yai) than the samples we have. Until fresh host-reared specimens of *Bactrocera irvingiae* can be obtained from the type locality and sequenced, we will defer from describing new species that may in the future turn out to be synonyms. Species 59 (Luang Nam Tha, Laos) and 60 (Jinghong, China) (Figure 15G–L) could not be definitely determined to species using available resources (Drew and Hancock 1994, Drew and Romig 2013), and did not match any of the OFF complex species examined in QDAF. They are likely new species, but not described here, due to the lack of distinctive characters and the very small number of specimens available (1 of species 59 and 3 of species 60). With additional survey work and genetic sequencing, a number of additional cryptic species likely will appear.

*Bactrocera dorsalis*, *Bactrocera invadens* and *Bactrocera papayae* were recently declared conspecific, and are genetically indistinguishable (Schutze et al. 2014a), despite what some consider diagnosable differences (Drew and Romig 2013). We have found a similar genetically indistinguishable situation for *Bactrocera osbeckiae*, *Bactrocera melastomatos* and *Bactrocera rubigina* (Wang and Zhao) in our phylogeny, despite them being very distinct from each other in color pattern (Figures 9F–O, 10, 14F). This suggests that these species, if distinct, may or may not differ at gene loci other than those sequenced in our study. *Bactrocera osbeckiae* and *Bactrocera rubigina* are sympatric in Thailand and Southern China (Leblanc, unpublished) and differ in color patterns and wing costal band expansion (Drew and Romig 2013), while *Bactrocera melastomatos* is confined to Peninsular Malaysia, Borneo, Java and Sumatra. *Bactrocera osbeckiae* and *Bactrocera melastomatos* are biologically close and peculiar, both breeding on flowers rather than fruits of Melastomataceae (Drew and Hancock 1994, Allwood et al. 1999), while the host range of *Bactrocera rubigina* is not well documented. Similarly, sympatric *Bactrocera tryoni* and *Bactrocera neohumeralis* in Australia are currently genetically inseparable, yet are likely valid biological species, isolated by time of mating (Clarke et al. 2011).

The high degree of intraspecific variation in color pattern severely limits the reliability of dichotomous and interactive keys. The range of variation differs considerably among species, with extreme cases like the scutum of *Bactrocera dorsalis* (Leblanc et al. 2013), and the abdomen of *Bactrocera thailandica* (Figure 13). Also, variants of unrelated species, such as *Bactrocera bhutaniae* (Figure 2) and *Bactrocera bivittata* (Figure 3), can overlap and make them hard to distinguish. The full extent of observed variation is easier to demonstrate in plates rather than words when describing a species. We suggest that descriptions of new species in the future should be accompanied by extensive plates showing variation, included in publications or posted as supplementary online material.

## Conclusion

The OFF complex was defined by Hardy (1969) and the definition refined by Drew and Hancock (1994). The species and specimens examined in this study fit their definition in all respects, except for scutum color, said to be mostly black (Drew and Hancock 1994) or black (Drew and Romig 2013). Several species included in the complex consistently have extensive pale markings on the scutum (e.g. *Bactrocera arecae* (Hardy and Adachi), *Bactrocera bivittata*, *Bactrocera cacuminata*, *Bactrocera osbeckiae*). *Bactrocera dorsalis* has a broad range of variation, from entirely black to extensively or almost entirely pale (Schutze et al. 2014b, Leblanc et al. 2013), a form that was described as the now-synonymized, *Bactrocera invadens* (Drew et al. 2005), which was not included in the OFF complex by Drew and Romig (2013). It is likely that at least some of the 21 other species complexes (Drew 1989, Drew and Romig 2013) are also polyphyletic and their morphological diagnostic characters not robust. Nonetheless, the *Bactrocera dorsalis* complex is likely to remain entrenched for some time in future literature, as an informal group referred to as a “collective group” in the International Code of Zoological Nomenclature (http://iczn.org/iczn/index.jsp). Caution must be exercised in literature to not refer to the group as a biological or evolutionary unit.

## Supplementary materials

Supplementary material 1 Figure S1 Data type: TIF File Format Explanation note: Maximum likelihood tree, based the *CO*I gene dataset. Support values above branches are Maximum Likelihood Bootstrap values / Bayesian Posterior Probabilities. Scale bar indicates the number of substitutions per site. Species in the Oriental fruit fly complex are outlined in red. This dataset is made available under the Open Database License (http://opendatacommons.org/licenses/odbl/1.0/). The Open Database License (ODbL) is a license agreement intended to allow users to freely share, modify, and use this Dataset while maintaining this same freedom for others, provided that the original source and author(s) are credited.Luc Leblanc, Michael San Jose, Norman Barr, Daniel Rubinoff

Supplementary material 2 Figure S2 Data type: TIF File Format Explanation note: Maximum likelihood tree, based the *EF-1α* gene dataset. Support values above branches are Maximum Likelihood Bootstrap values / Bayesian Posterior Probabilities. Scale bar indicates the number of substitutions per site. Species in the Oriental fruit fly complex are outlined in red. This dataset is made available under the Open Database License (http://opendatacommons.org/licenses/odbl/1.0/). The Open Database License (ODbL) is a license agreement intended to allow users to freely share, modify, and use this Dataset while maintaining this same freedom for others, provided that the original source and author(s) are credited.Luc Leblanc, Michael San Jose, Norman Barr, Daniel Rubinoff

Supplementary material 3 Figure S3 Data type: TIF File Format Explanation note: Maximum likelihood tree, based the *period* gene dataset. Support values above branches are Maximum Likelihood Bootstrap values / Bayesian Posterior Probabilities. Scale bar indicates the number of substitutions per site. Species in the Oriental fruit fly complex are outlined in red. This dataset is made available under the Open Database License (http://opendatacommons.org/licenses/odbl/1.0/). The Open Database License (ODbL) is a license agreement intended to allow users to freely share, modify, and use this Dataset while maintaining this same freedom for others, provided that the original source and author(s) are credited.Luc Leblanc, Michael San Jose, Norman Barr, Daniel Rubinoff
