# Supplementary figures and images for: A phylogenetic assessment of the polyphyletic nature and intraspecific color polymorphism in the Bactrocera dorsalis complex (Diptera, Tephritidae)
Source: Zookeys. 2015 Nov 26;(540):339–67. doi: 10.3897/zookeys.540.9786 (PMC4714077; doi:10.3897/zookeys.540.9786)

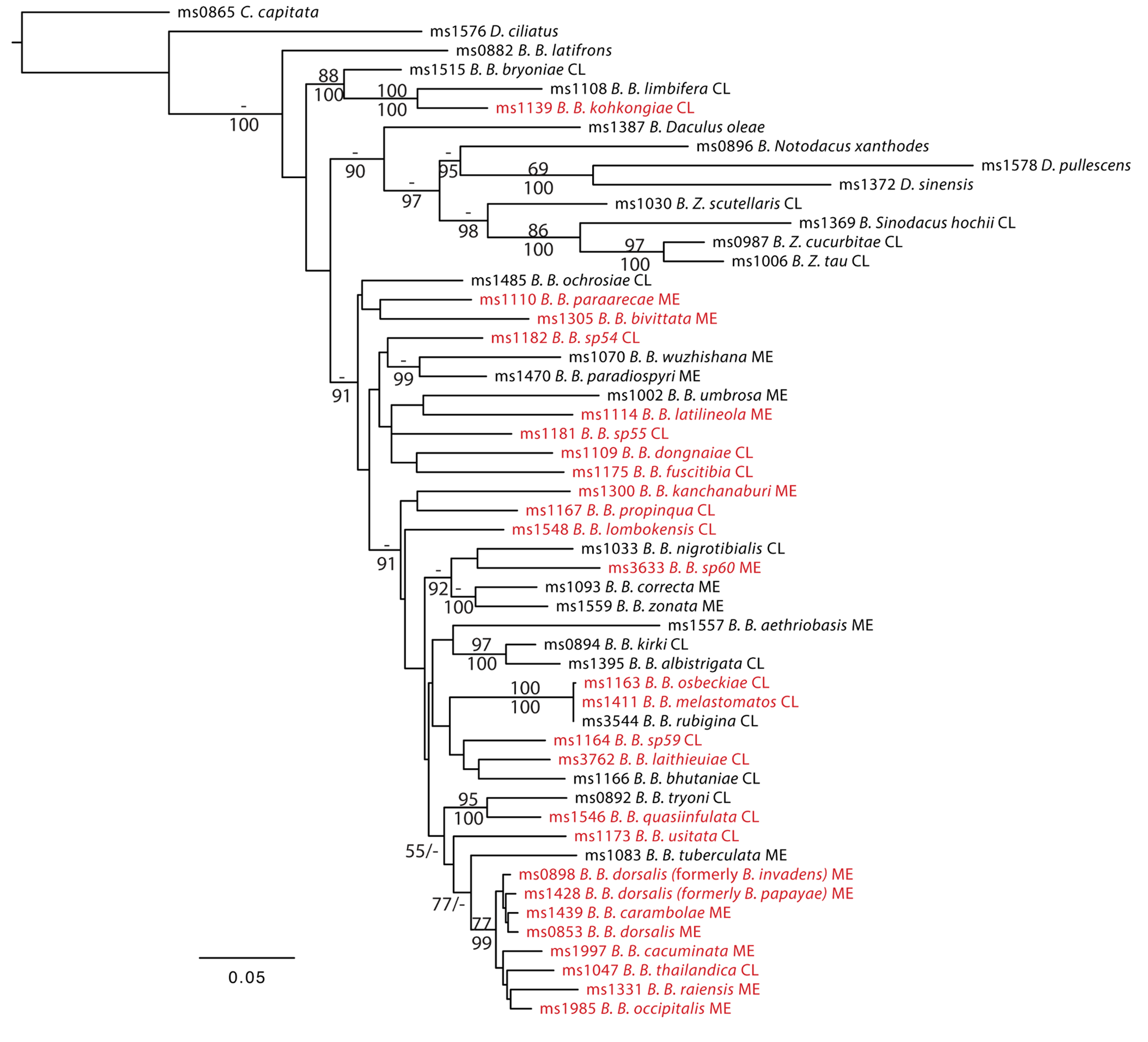

Supplement: Supplementary material 1 — Figure S1 [file zookeys-540-339-s001.tif]

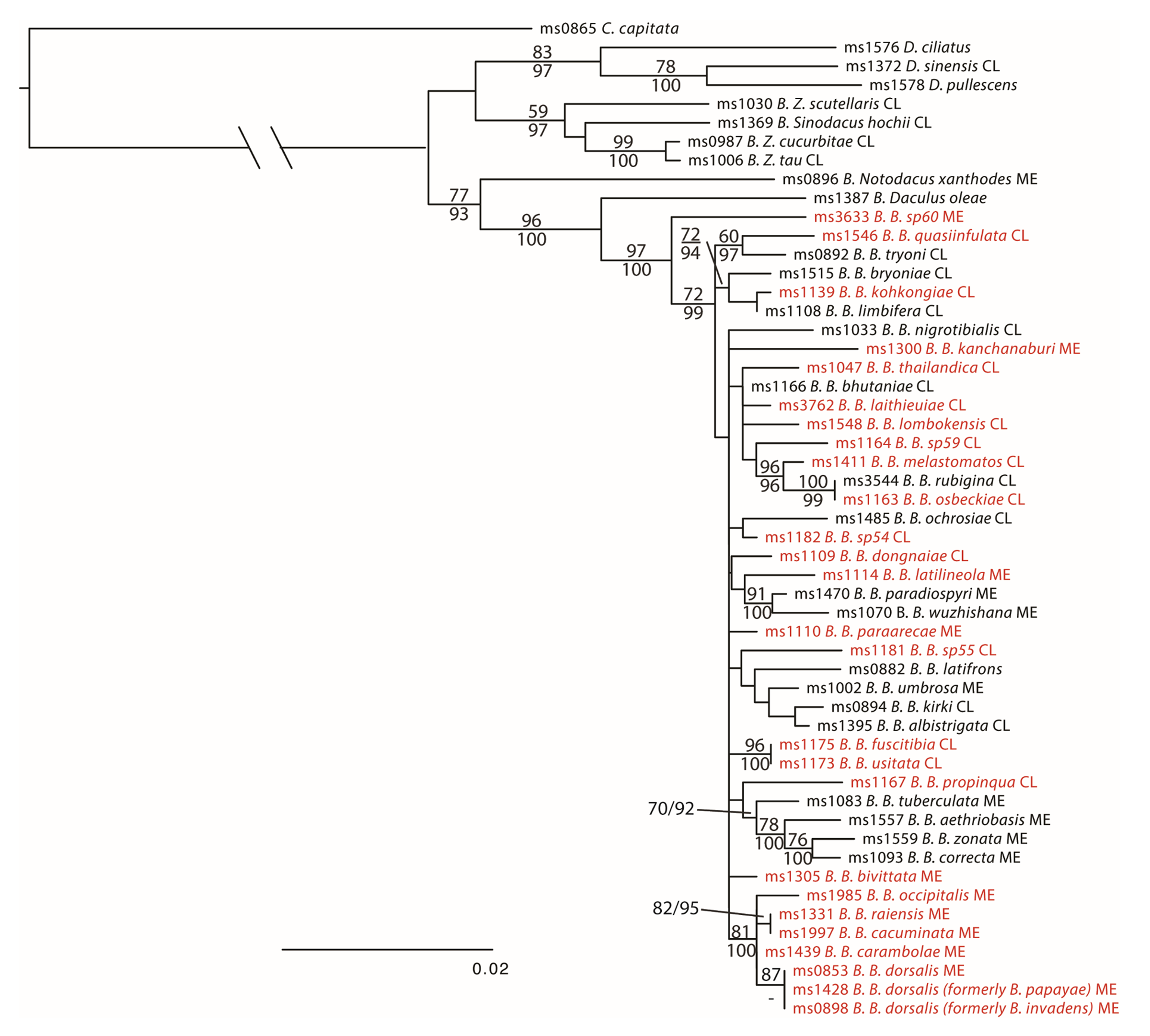

Supplement: Supplementary material 2 — Figure S2 [file zookeys-540-339-s002.tif]

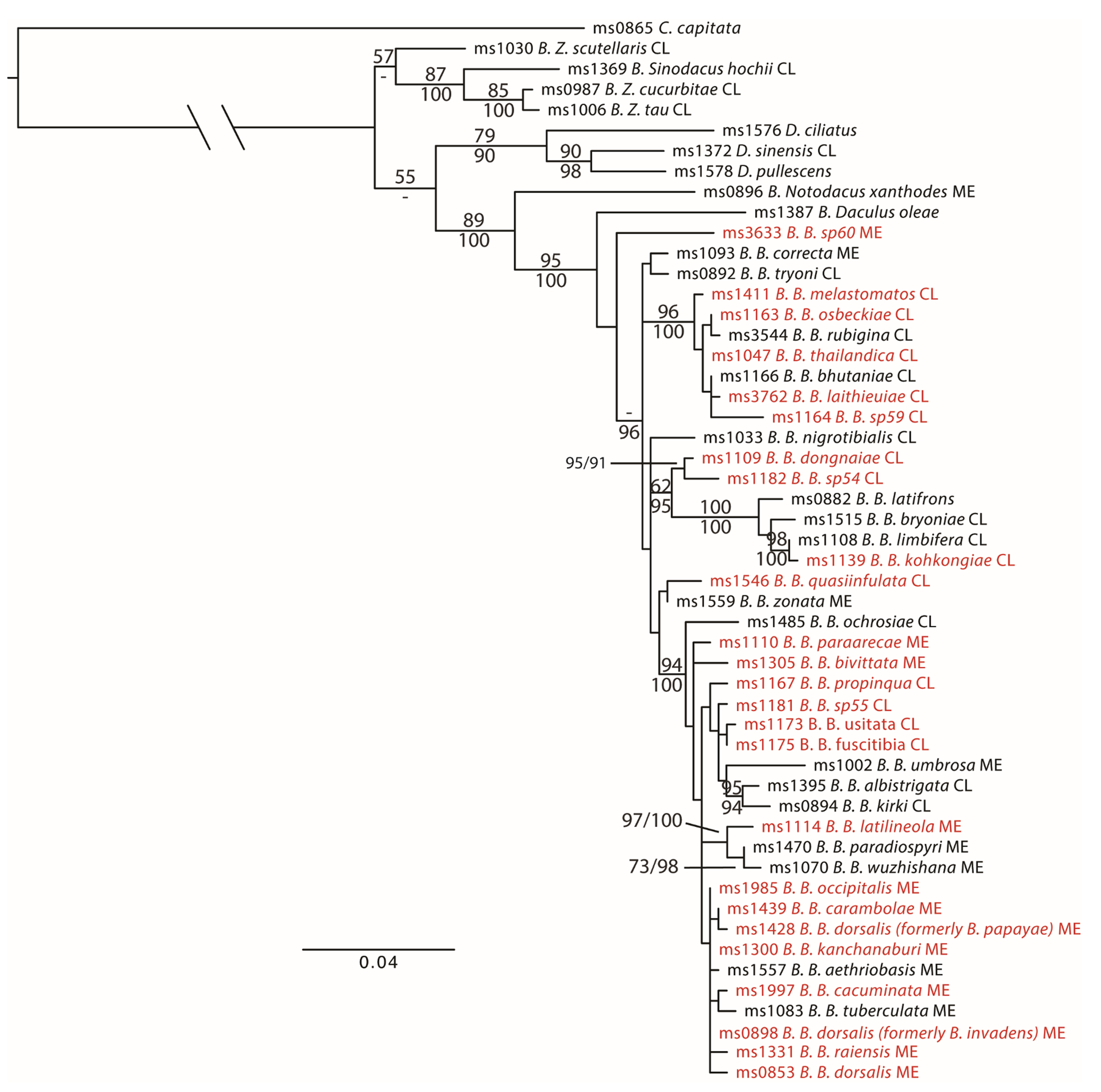

Supplement: Supplementary material 3 — Figure S3 [file zookeys-540-339-s003.tif]
